# Biomonitoring California Protocol for Following up on Elevated Levels of Urinary Arsenic

**DOI:** 10.3390/ijerph20075269

**Published:** 2023-03-27

**Authors:** Shoba Iyer, Duyen Kauffman, Craig Steinmaus, Sara Hoover

**Affiliations:** 1 Office of Environmental Health Hazard Assessment (OEHHA), Oakland, CA 94612, USA; 2 San Francisco Environment Department (SFE), San Francisco, CA 94103, USA; 3 California Department of Public Health (CDPH), Richmond, CA 94804, USA; 4 Superfund Research Program, University of California, Berkeley, CA 94720, USA

**Keywords:** biomonitoring, arsenic

## Abstract

Objectives: to develop and implement a follow-up protocol for Biomonitoring California study participants with elevated levels of urinary arsenic, particularly inorganic forms. Methods: We selected 20 μg/L as the level of concern for urinary *inorganic* arsenic; samples with *total* arsenic ≥20 μg/L were speciated. Participants with elevated inorganic arsenic were notified of their level and invited to participate in a telephone survey to help determine possible exposure sources. We illustrate the protocol in four Biomonitoring California studies, which collected samples from 2010–2013 in locations across the state. Results: 48 participants in the four studies had elevated urinary inorganic arsenic levels. Consumption of rice and rice-based products was the most commonly identified potential source of inorganic arsenic exposure. Conclusions: Of 48 participants with elevated inorganic arsenic, 27 would have been missed if we had used the previously published threshold of 50 µg/L total arsenic to identify urine samples for speciation. This protocol fills a gap in the clinical literature by providing a more health-protective approach to identify individuals with elevated urinary inorganic arsenic and help determine potentially significant exposure sources.

## 1. Introduction

Biomonitoring California is a legislatively mandated program that measures and tracks levels of selected environmental chemicals in people. Chemicals can be chosen for biomonitoring studies from the program’s list of designated chemicals [1], which includes metals, perfluoroalkyl and polyfluoroalkyl substances, phenols, quaternary ammonium compounds, and many other chemical groups. The primary focus of these studies is to develop robust chemical exposure data, which can inform research by other groups exploring the linkages between these exposures and health. Biomonitoring California study results also help guide and evaluate the State’s efforts to reduce specific chemical exposures.

The enabling legislation, signed into law in 2006, requires trained program staff to consult with study participants and recommend follow-up steps if their biomonitoring results indicate a significant known health risk [2]. To guide this process, Biomonitoring California’s Scientific Guidance Panel advised the program to adopt biological levels determined by state or federal agencies to be of concern (which we deem “levels of concern [LOCs]”). Of the chemicals we measure, we have identified LOCs for *total* arsenic (urine), cadmium (blood and urine), lead (blood), and mercury (blood and urine). Total arsenic reflects both inorganic and organic forms; however, the inorganic form is of most concern for human health. We therefore developed a practical approach to identify and follow up on elevated *inorganic* urinary arsenic levels, in addition to total arsenic.

Inorganic arsenic compounds are listed under California’s Proposition 65 (Title 27, California Code of Regulations, § 27001) as known to cause cancer and reproductive toxicity (developmental endpoint) [3]. Human exposure to inorganic arsenic is also linked with other health effects, such as cardiovascular disease [4,5,6,7,8] and neurotoxicity [9,10]. Inorganic arsenic has been detected in some groundwater sources [11], public drinking water [12], and cropland soils [13] in California, making it an ongoing public health concern for the state. The Centers for Disease Control and Prevention (CDC) established a case definition for inorganic arsenic poisoning, which recommends speciation of urine samples with total arsenic levels > 50 μg/L but does not address inorganic arsenic levels [14]. We adopted this value as our program’s LOC for total urinary arsenic. We did not identify an established LOC for urinary inorganic arsenic in the general population, nor an applicable urinary threshold for the toxic effects of inorganic arsenic from the scientific literature [15]. Caldwell et al. [16] statistically determined a “cut-point” of 20 µg/L for urinary inorganic arsenic, which we selected as the LOC for inorganic arsenic. Any participants with urinary inorganic arsenic at or above 20 µg/L are considered to have elevated levels. We use 20 µg/L total urinary arsenic to identify urine samples for speciation to ensure that all samples with potentially elevated inorganic arsenic are tested. This is a more health-protective approach than using the existing guidance value of 50 μg/L for total arsenic, while still practical in focusing on the most highly exposed participants for required follow-up. We also conducted literature research to develop a detailed survey about potentially important sources of inorganic arsenic exposures for impacted participants. Here, we discuss the results of applying this protocol in four example studies.

## 2. Methods

### 2.1. Biomonitoring California Studies

We illustrate the development and application of this protocol in four Biomonitoring California studies: the Maternal and Infant Environmental Exposure Project (MIEEP) [17], the Firefighter Occupational Exposures (FOX) Project [18,19], the Pilot Biomonitoring Exposures Study (PBEST) [20], and Expanded BEST (EBEST) [21]. Study participants included Californians who may be particularly vulnerable to the effects of inorganic arsenic (e.g., pregnant women). Urine samples were collected and analyzed for arsenic, as part of a metals panel [22], during the years 2010 to 2013. For MIEEP, FOX, and PBEST, the Environmental Health Laboratory (EHL) in the California Department of Public Health (CDPH) (one of Biomonitoring California’s laboratories) conducted the arsenic testing and speciation [23]. For EBEST, Brooks Applied Labs (https://brooksapplied.com/, accessed on 13 March 2023) conducted the testing to implement the arsenic protocol, due to limited program laboratory capacity at that time. All arsenic testing is now being conducted by EHL. We adhered to study protocols approved by the relevant Institutional Review Boards. More information on these example studies, including the other analytes measured, is available on the Biomonitoring California website [24] and in selected Program publications [25].

### 2.2. Development of Elevated Arsenic Protocol

#### 2.2.1. Levels of Concern for Urinary Arsenic

Biomonitoring California’s LOC for total urinary arsenic is ≥50 µg/L. We adopted the Caldwell et al. cut point of 20 µg/L as the LOC for urinary inorganic arsenic, which they defined as the sum of the metabolites dimethylarsinic acid (DMA), monomethylarsonic acid (MMA), arsenic (V) acid, and arsenous (III) acid. This corresponded roughly to the 95th percentile for a random one-third subsample of all participants (age ≥ 6 years) in the National Health and Nutrition Examination Survey (NHANES) 2003–2004 [16]. To ensure that we capture all participants with urinary inorganic arsenic above the LOC, we use 20 µg/L total arsenic to identify samples for speciation.

#### 2.2.2. Notification

As mandated by our enabling legislation, individual biomonitoring results are made available to study participants. The results packet includes fact sheets on the measured chemicals and other resources. For the four studies discussed here, participants with urinary inorganic arsenic levels ≥20 µg/L were initially contacted about their elevated results via a telephone call. As a health-protective measure, we also contacted one EBEST participant with 19.2 µg/L inorganic arsenic. For interested participants, the telephone survey on arsenic exposures (see Section 2.2.3) was administered to help determine potential sources. Possible ways to reduce exposures were also discussed. Notification letters were mailed to all participants with urinary inorganic arsenic levels ≥20 µg/L, regardless of whether phone contact was successful. These letters included the speciated arsenic results and a fact sheet [26] with possible ways to reduce exposures; this same information was provided again in the complete results packet. For participants who had not been reached by telephone, the letters included an invitation to contact us for administration of the voluntary survey.

Participants with total arsenic levels ≥ 50 µg/L *and* inorganic arsenic levels < 20 µg/L received a notification letter that their elevated urinary total arsenic levels were likely attributable to arsenobetaine from recent seafood consumption. The letter explained that this form of arsenic is not considered a health concern.

Participants with concerns or questions about their arsenic levels were offered the opportunity to speak with Dr. Craig Steinmaus, an arsenic expert and Public Health Medical Officer at the Office of Environmental Health Hazard Assessment (OEHHA).

#### 2.2.3. Telephone Survey for Arsenic Exposures

Biomonitoring California study participants provide information on demographics and potential chemical exposures via surveys administered in person or online as part of the standard protocol. The goal of the additional telephone survey described here was to help identify arsenic exposure sources for participants with elevated urinary levels and address any concerns or questions related to their results. We based this survey on the arsenic poisoning case report form developed by the Florida Department of Health [27] and expanded it to include questions about potentially important exposure sources gleaned from ongoing comprehensive literature searches. The additional questions covered consumption of rice and rice-based foods [28,29,30], hijiki seaweed [31], mushrooms [32,33], and some types of juice [34,35]; participants’ travel (e.g., to an international location) in the week prior to providing their urine sample; nutritional supplement intake [36]; and other newly identified sources of arsenic. An example of the telephone survey is provided as a Supplemental File. Trained staff administering the survey deviated from the script as needed to clarify participants’ answers or respond to questions.

### 2.3. Application of Elevated Arsenic Protocol

**Figure 1:**
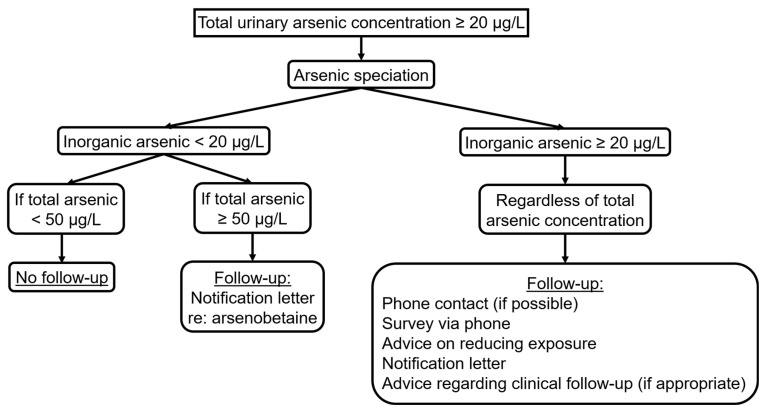
Flow chart of protocol for evaluating and following up on urinary arsenic levels.

Figure 1 shows how the protocol was applied.

For the four studies discussed here, the telephone survey was administered 1.5 to 3 years following the collection of the urine samples. The delay was primarily due to lengthy recruitment periods and limited laboratory capacity. We informed participants that the survey was voluntary and that they could refuse to take it, skip questions, or stop at any time.

One survey question focused on the water source(s) used for drinking and cooking. For PBEST and EBEST, we also obtained average arsenic concentrations in local water systems from data used for the drinking water indicator in OEHHA’s CalEnviroScreen tool [37].

## 3. Results

Table 1 provides brief descriptions of the four studies discussed in this paper. It also includes the numbers of urine samples analyzed for total arsenic; the subsets that were speciated; the samples with inorganic arsenic levels ≥20 µg/L; and response rates for the telephone survey.

**Figure 2:**
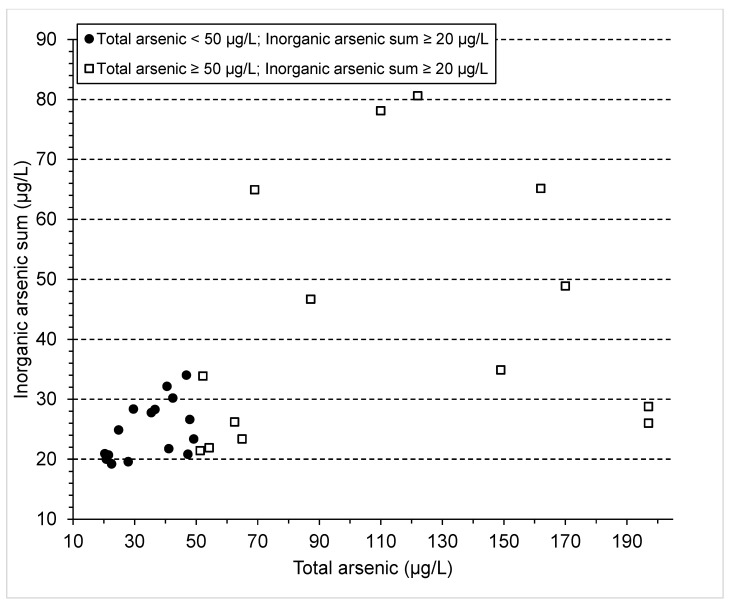
Urinary inorganic arsenic levels (µg/L) are plotted against total urinary arsenic (µg/L) for EBEST participants with elevated inorganic arsenic levels (*n* = 30). The filled circles represent participants’ urine samples with total urinary arsenic <50 µg/L and inorganic arsenic ≥20 µg/L. The unfilled squares represent participants’ urine samples with total urinary arsenic ≥50 µg/L and inorganic arsenic ≥20 µg/L.

Results from the 30 EBEST participants with elevated urinary levels of inorganic arsenic are plotted in Figure 2. Urinary inorganic arsenic levels ranged from 19.2 to 80.6 µg/L. Twenty-nine EBEST participants had levels of inorganic arsenic ≥ 20 µg/L. Fifteen of these would have been missed if we had speciated only the samples with total arsenic ≥50 µg/L instead of ≥20 µg/L.

The majority of EBEST participants (27/30) with elevated inorganic arsenic levels elected to take the telephone survey to help determine their potential exposure sources. Only one of the three remaining participants declined the survey; the other two could not be reached within our IRB-approved limit of three calls. Table 2 displays some potential contributing sources of arsenic exposures identified from their responses and the supplementary drinking water information. Consumption of rice and rice-based products was the most common potential contributor to elevated inorganic arsenic, with seafood consumption likely responsible for elevated organic arsenic. Other possible sources of inorganic arsenic were beer, wine, and/or sake; hijiki seaweed; occupational exposures (see Table 2); and drinking water.

The survey responses we obtained from FOX and PBEST participants identified similar potential arsenic exposure sources. Both FOX survey respondents reported consumption of rice and rice-based products as well as fish and/or shellfish. The three PBEST survey respondents also reported consumption of rice and rice-based products as well as fish and/or shellfish. Other possible inorganic arsenic sources identified for these PBEST participants included beer, wine, and/or sake consumption, and occupational exposures (e.g., employment at a glass manufacturing plant; contact with pressure-treated wood).

Participants’ reactions to learning about their elevated arsenic levels varied. In general, they were curious to learn more about arsenic and its sources, and in most cases they were not overly concerned. Some participants expressed concerns about health effects, the potential for ongoing exposures, and similar exposures for family members. Several participants questioned the relevance of results that were several years old and requested follow-up testing for themselves and others in their household, the latter of which was not possible under the study protocols. Given the large number of EBEST participants affected by the delay in arsenic speciation, we made special arrangements for participants who expressed interest in follow-up testing (25/30) to collect and ship additional urine samples. Due to logistics and other complications, only 15 of the 25 participants sent their urine samples for repeat analyses. These 15 participants all took the associated exposure survey, showing a high motivation to learn about their potential exposure sources.

## 4. Discussion

We developed a practical approach for identifying and following up with participants who had elevated urinary arsenic levels and illustrated its application in four Biomonitoring California studies. We chose 20 µg/L as the inorganic arsenic LOC, based on Caldwell et al. This is a statistically determined cut-point approximately corresponding to the 95th percentile of urinary inorganic arsenic from NHANES 2003–2004 for a subsample of all participants (age ≥ 6 years) [16]. We examined the 95th percentile for urinary inorganic arsenic reported by CDC for random subsamples of all participants in subsequent NHANES cycles [38]. It initially declined through the 2007–2008 cycle (16.8 µg/L), rising again in 2009–2010 (20.8 µg/L). Since then, it has steadily dropped (2011–2012: 17.2 µg/L; 2013–2014: 14.7 µg/L; 2015–2016: 14.5 µg/L; 2017–2018: 13.4 µg/L). Younger children (3–5 years) were included in the two most recent cycles. We have retained the Caldwell et al. [16] cut-point of 20 µg/L as the inorganic arsenic LOC in the Biomonitoring California protocol, but the program could reevaluate this in the future, particularly if a clinically based value is established by CDC or other agencies.

We speciate all urine samples with total urinary arsenic ≥20 µg/L. This health-conservative approach identified a total of 27 participants in the four example studies with elevated inorganic arsenic whose total arsenic levels were less than 50 µg/L. If we had speciated only the samples with total arsenic ≥ 50 µg/L, these participants would not have received important follow up on their potentially harmful exposures to inorganic arsenic.

The telephone surveys consistently identified rice and rice-based products as a potential source of inorganic arsenic exposures for the PBEST, EBEST, and FOX participants with whom we were able to follow up. This is in line with information reported in other publications [28,39,40].

Our findings on likely arsenic sources are limited by the small numbers of participants who completed the telephone survey in the example studies we chose to illustrate this protocol. The highest response rate was in EBEST. We attribute this in part to having current telephone numbers for almost all participants, because KPNC, our study partner, routinely updates members’ contact information. MIEEP participants were particularly difficult to reach, partially due to challenges in obtaining reliable contact information. Records for these prenatal patients were closed and stored by SF General after childbirth and were inaccessible to Biomonitoring California staff.

Following Caldwell et al. [16], our protocol defines urinary inorganic arsenic as the sum of DMA, MMA, arsenic (V) acid, and arsenous (III) acid. However, DMA is not exclusively a metabolite of inorganic arsenic. There are some types of fish and shellfish that can contain high levels of organic arsenosugars and arsenolipids, which are metabolized by humans to DMA [41,42]. Therefore, our identification of elevated urinary inorganic arsenic for some participants could instead reflect DMA exposure from recent consumption of fish and shellfish. Our protocol uses one-on-one consultation with each participant to address the possible identification of some samples as having high inorganic arsenic when the source is more likely DMA from seafood consumption.

Some seafood might be actual sources of inorganic arsenic (e.g., some clams and crabs [43]). However, we had insufficient information to determine how important this is for seafood consumed in California.

Extended study recruitment periods and limitations in laboratory capacity resulted in a time lag between participants’ urine sample collection and the administration of the telephone survey, particularly for EBEST. Given this delay, participants could have had difficulty in recalling information relevant to possible sources of inorganic arsenic exposures. For more recent program studies, the time lag has been reduced. For example, in the California Regional Exposure (CARE) Study, participants were offered the follow-up telephone survey within one month of identifying urine samples with elevated arsenic levels, which was typically within eight months of sample collection. CARE Study participants were also asked questions related to potential arsenic exposures at the time of urine sample collection, which improved data on likely sources.

## 5. Conclusions

Our protocol is a practical approach for identifying and following up with Biomonitoring California participants who are most highly exposed to arsenic, using speciation to evaluate their levels of urinary inorganic forms. The detailed telephone survey helps identify potentially important sources of inorganic arsenic exposures for these participants. We conduct individual consultations to delve into their specific exposures and provide an opportunity for them to ask questions and have their concerns addressed. We also explain that elevated arsenic levels linked to seafood consumption are not considered to be of concern. Setting 20 µg/L as the total urinary arsenic level to identify samples for speciation is a more health-protective approach than previously published guidance (i.e., 50 µg/L total arsenic). Choosing this lower level to screen samples is warranted by the serious health concerns associated with exposure to inorganic arsenic.

## Figures and Tables

**Table 1:** Overview of four Biomonitoring California studies.

Study	Description	Number of Urine Samples Analyzed		Number of Samples with Inorganic Arsenic ≥20 µg/L (*n*)	Response Rate of Participants for Telephone Survey
		Total Arsenic (*n*)	Speciated Arsenic (*n*)		
Maternal and Infant Environmental Exposure Project (MIEEP)	Pregnant women were recruited at San Francisco (SF) General Hospital. The participants were primarily Hispanic. Urine samples were collected during the third trimester of pregnancy in 2010–2011.	89	13	6	0/6
Firefighter Occupational Exposures (FOX) Project	A convenience sample of firefighters was recruited from a Southern California county. The firefighters were predominantly non-Hispanic white males. Urine samples were collected in 2010–2011.	101	29	4	2/4
Pilot Biomonitoring Exposures Study (PBEST)	Adult members of Kaiser Permanente Northern California (KPNC) living in California’s Central Valley were recruited using a stratified random sampling design (i.e., age, gender, race/ethnicity, location). Urine samples were collected in 2011–2012.	111	29	8	3/8
Expanded BEST (EBEST)	Adult KPNC members living in California’s Central Valley were recruited using a stratified random sampling design that put a special emphasis on sampling of Hispanics and Asian/Pacific Islanders. Urine samples were collected in 2013.	218	57	30 ^a^	27/30

^a^ This includes one urine sample with 19.2 µg/L inorganic arsenic, which was speciated as a health-protective measure. We have incorporated results for this participant in Figure 2 and the description in Section 3.

**Table 2:** Potential sources of arsenic exposures for EBEST telephone survey respondents.

Potential Exposure Source ^a^	Number of EBEST Survey Respondents with this Exposure (total *n* = 27)	Comments
Rice and rice-based products	25	Regularly consumed ^b^ rice and rice-based products
Hijiki seaweed	4	Indicated any intake of hijiki seaweed
Fish and/or shellfish ^c^	23	Regularly consumed fish and/or shellfish
Beer, wine, and/or sake (rice wine)	10	Regularly consumed beer, wine, and/or sake
Occupational exposures	5	Worked with electronics and batteries, pressure-treated wood, or chemicals at a facility for manufacturing glass bottles and bottling wine
Drinking water	5 ^d^	Lived in areas where average arsenic concentration in water system is greater than the Maximum Contaminant Level of 10 ppb

^a^ Consumption of fish and/or shellfish likely contributes to organic arsenic exposure; the remaining sources listed here are potential sources of inorganic arsenic. Other survey questions potentially relevant to inorganic arsenic exposures included contact with soil; cigarette smoking; taking dietary supplements/vitamins or herbal medicines/traditional remedies, particularly imported ones; travel just prior to providing the urine sample; and dietary changes since providing the urine sample. These did not reveal any additional clear contributors to urinary inorganic arsenic exposure. ^b^ “Regularly consumed” means survey respondents reported consumption of an item at least once in the last three days or at least once a week, or reported that they usually eat or drink the item. ^c^ Arsenobetaine is the predominant type of arsenic in fish and shellfish and is generally not considered a health concern. ^d^ One additional participant reported working at a site known to have arsenic in the drinking water at the time of donating a urine sample.

## Data Availability

Data are available upon request from the corresponding author, except when release of the data would compromise study participant identity.

## Supplementary Materials

The following is available online at https://www.mdpi.com/article/10.3390/ijerph20075269/s1, Supplemental File: Telephone Survey for Arsenic in Adult Participants from Biomonitoring California Projects.
