# Supplementary material for: Biomonitoring California Protocol for Following up on Elevated Levels of Urinary Arsenic
Source: Int J Environ Res Public Health. 2023 Mar 27;20(7):5269. doi: 10.3390/ijerph20075269 (PMC10094481; doi:10.3390/ijerph20075269)
Supplement: Supplementary file 1 [file ijerph-20-05269-s001.zip › ijerph-2204141-supplementary.pdf]

Biomonitoring California Protocol for Following Up on Elevated Levels of Urinary Arsenic

Supplemental File

TELEPHONE SURVEY FOR ARSENIC IN ADULT PARTICIPANTS FROM BIOMONITORING CALIFORNIA PROJECTS[1]

Participant ID#

Date survey completed:____/_____/______(mo/day/yr)

Survey administered by_________________(first name) ______________________(last name)

Refer to telephone script for how to initiate contact or leave a message if necessary.

As part of the Biomonitoring Exposures Study (BEST), we collected a urine sample from you on <insert date> and measured various chemicals, including arsenic. Arsenic is found in soil, water and some foods.

We found that the level of total arsenic in your urine was below the level of concern, but somewhat higher than expected. We did additional testing to learn more about the specific forms of arsenic in your urine, because that helps us understand the possible sources of your exposures. Your result for the form of arsenic called “inorganic arsenic” was above the level of concern for that specific form. The level of concern for inorganic arsenic is based on levels seen in the general U.S. population. I would like to ask you some questions that will help us understand how you may have been exposed to inorganic arsenic. This is a voluntary survey. You can refuse to take the survey, you can skip any of the questions, or you can stop at any time. Do you have any questions for me before we begin?

____

____

____

____

____

____

[1] Some questions in this survey were drawn from the Florida Department of Health Acute Arsenic Poisoning Case Report Form. (http://www.floridahealth.gov/diseases-and-conditions/disease-reporting-and-management/disease-reporting-and-surveillance/_documents/crf-arsenic.pdf)

I'll start by asking questions about your diet, because certain foods and beverages can contain arsenic.

1. Do you eat rice?
   ☐ Yes
   - If Yes:
     - How many times in the last 3 days have you eaten rice?______________________
       ☐ Don't know / unsure
       ☐ Refused
     - About how much rice did you eat each time?
       ____
       ☐ Don't know / unsure
       ☐ Refused
     - Can you tell me the brand name and type (white, brown, etc.) of rice that you ate?
       ____
       ☐ Don't know / unsure
       ☐ Refused

   ☐ No
   ☐ Don't know / unsure
   ☐ Refused

2. Do you eat or drink rice-based products? Some examples include cold rice cereals (such as Rice Krispies®), hot rice cereals (such as Cream of Rice®), rice cakes, rice crackers, rice pasta or noodles, rice pudding, brown rice syrup, rice milk, and horchata (a drink that can be made from rice). (*Note to staff administering survey – ask follow-up questions on serving sizes as needed and fill out data table for question 2.*)
   ☐ Yes
   - If Yes:
     - Can you tell me the types and brand names of the rice-based products you ate or drank in the last 3 days?
       ____
       ☐ Don't know / unsure
       ☐ Refused
     - For each rice-based product, how many times did you consume it as a meal or snack in the last 3 days?____
       ☐ Don't know / unsure
       ☐ Refused
     - For each rice-based product, about how much did you consume each time you ate or drank it?
       ____
       ☐ Don't know / unsure
       ☐ Refused

   ☐ No
   ☐ Don't know / unsure
   ☐ Refused

3. Do you eat prepared foods that list brown rice syrup as an ingredient? Some examples of foods that may contain brown rice syrup are energy bars, cereal bars, granola bars, and granola. (*Note to staff administering survey – ask follow-up questions on serving sizes as needed and fill out data table for question 3.*)
   ☐ Yes
   - If Yes:
     - Can you tell me the types and brand names of the foods that may contain brown rice syrup that you ate in the last 3 days?

       ____
       ☐ Don't know / unsure
       ☐ Refused
     - For each food item that may contain brown rice syrup, how many times did you consume it as a meal or snack in the last 3 days?

       ____
       ☐ Don't know / unsure
       ☐ Refused
     - For each food item that may contain brown rice syrup, about how much did you consume each time you ate it?

       ____
       ☐ Don't know / unsure
       ☐ Refused

   ☐ No
   ☐ Don't know / unsure
   ☐ Refused

4. Do you, or does someone in your home, prepare or cook foods using rice-based ingredients, such as brown rice syrup or rice wine vinegar? *(Note to staff administering survey - see table for question 4 at the end of this questionnaire to fill in responses.)*
   ☐ Yes
   - If Yes:
     - Can you tell me the types of foods containing rice-based ingredients that you ate in the last 3 days?

       ____
       ☐ Don't know / unsure
       ☐ Refused
     - Can you tell me the brands of rice-based ingredients used in these foods?

       ____
       ☐ Don't know / unsure
       ☐ Refused
     - For each food item prepared in your home using a rice-based ingredient, how many times did you consume it as a meal or snack in the last 3 days?

       ____
       ☐ Don't know / unsure
       ☐ Refused
     - For each food item prepared using a rice-based ingredient, about how much did you consume each time you ate or drank it?

       ____
       ☐ Don't know / unsure
       ☐ Refused

   ☐ No
   ☐ Don't know / unsure
   ☐ Refused

5. Do you eat seaweed in your usual diet?
   ☐ Yes
   - If Yes:
     - How many times did you eat seaweed in the last 3 days?
       ____
       ☐ Don't know / unsure
       ☐ Refused
     - Hijiki seaweed is a short, black, noodle-like seaweed. How many times did you eat this type of seaweed in the last 3 days?
       ____
       ☐ Don't know / unsure
       ☐ Refused
     - About how much hijiki seaweed did you eat each time?
       ____
       ☐ Don't know / unsure
       ☐ Refused

   ☐ No
   ☐ Don't know / unsure
   ☐ Refused

6. The type of arsenic found in fish and shellfish is not considered to be a health concern; knowing if you eat these foods helps us interpret your arsenic results. Did you eat fish or shellfish in the last week?
   ☐ Yes
   - If Yes:
     - How many of your meals in the last week contained fish or shellfish?
       ____
       - ☐ Don't know / unsure
         ☐ Refused
     - What kinds of fish or shellfish did you eat?
       ____
       ☐ Don't know / unsure
       ☐ Refused
     - About how much fish or shellfish did you eat each time?
       ____
       - ☐ Don't know / unsure
         ☐ Refused

   ☐ No (skip to question 8)
   ☐ Don't know / unsure
   ☐ Refused

7. Did you eat fish or shellfish caught by you, family members, or friends in the last week?
   ☐ Yes
   - How many of your meals in the last week contained fish or shellfish caught by you, family members, or friends?
     ____
     - ☐ Don’t know / unsure
       ☐ Refused
   - Where was the fish caught?
     ____
   - What kinds of fish or shellfish did you eat?
     ____
     ☐ Don’t know / unsure
     ☐ Refused
   - About how much fish or shellfish did you eat each time?
     ____
     - ☐ Don’t know / unsure
       ☐ Refused

   ☐ No
   ☐ Don’t know / unsure
   ☐ Refused

8. Some studies report finding arsenic in mushrooms. Do you eat mushrooms, including store-bought mushrooms or wild mushrooms that you collect? (*Note to staff administering survey – ask follow-up questions on foraging locations and mushrooms collected as needed and fill out data table for question 8.*)
   ☐ Yes
   - If Yes:
     - How many times in the last 3 days have you eaten mushrooms?________________
       ☐ Don’t know / unsure
       ☐ Refused
     - When you ate mushrooms in the last 3 days, about how much did you eat each time?
       ____
       ☐ Don’t know / unsure
       ☐ Refused
     - What particular type or types of mushrooms did you eat?
       ____
       ☐ Don’t know / unsure
       ☐ Refused
     - Do you forage for mushrooms?
       ☐ Yes
       - If Yes – in which locations do you forage for mushrooms? For each location, can you tell me what type or types of mushrooms you collect there?____
       ☐ No
       ☐ Don’t know / unsure
       ☐ Refused

   ☐ No
   ☐ Don’t know / unsure
   ☐ Refused

9. This question is about alcoholic beverages. Arsenic has been found in some alcoholic beverages. Did you drink beer, wine, and/or sake (rice wine) in the last 3 days? (*Note to staff administering survey – ask follow-up questions on serving sizes as needed and fill out data table for question 9.*)
   ☐ Yes
   - If Yes:
     - Can you tell me the types and brand names of the alcoholic beverages you drank in the last 3 days?

       ____

       ☐ Don't know / unsure
       ☐ Refused
     - For each beverage type (beer, wine, and/or sake [rice wine]), how many times did you drink it in the last 3 days?

       ____

       ☐ Don't know / unsure
       ☐ Refused
     - For each beverage type (beer, wine, and/or sake [rice wine]), about how much did you drink each time?

       ____

       ☐ Don't know / unsure
       ☐ Refused

   ☐ No
   ☐ Don't know / unsure
   ☐ Refused

10. There are reports of arsenic in some teas. Do you drink tea? (*Note to staff administering survey – ask follow-up questions on serving sizes as needed and fill out data table for question 10.*)
   ☐ Yes
   - If Yes:
     - What types of tea (e.g., black, green, herbal) did you drink in the last 3 days?

       ____

     - Can you tell me the brand names (such as Lipton®, Twinings®, Celestial Seasonings®) or specific types of these teas (such as English breakfast, Assam black tea, rooibos), and/or what country they came from?

       ____

       ☐ Don't know / unsure
       ☐ Refused
     - For each type of tea, how many times did you drink it in the last 3 days?

       ____

       ☐ Don't know / unsure
       ☐ Refused
     - For each type of tea, about how much did you drink each time?

       ____

       ☐ Don't know / unsure
       ☐ Refused

   ☐ No
   ☐ Don't know / unsure
   ☐ Refused

11. Arsenic has been found in a few kinds of apple and grape juices. Do you drink apple and/or grape juice? *(Note to staff administering survey - see table for question 11 at the end of this questionnaire to fill in responses.)*

☐ Yes
- If Yes:
  - What types of apple or grape juice did you drink in the last 3 days? ________________________________
  - Can you tell me the brand names of these juices? ________________________________
    ☐ Don't know / unsure
    ☐ Refused
  - For each type of juice (apple and grape), how many times did you drink it in the last 3 days?________________________________
    ☐ Don't know / unsure
    ☐ Refused
  - For each type of juice (apple and grape), about how much did you drink each time? ________________________________
    ☐ Don't know / unsure
    ☐ Refused

☐ No
☐ Don't know / unsure
☐ Refused

12. Do you drink or cook with water that comes from a private well?

☐ Yes ☐ No ☐ Don't know / unsure ☐ Refused
- If Yes – has the water been tested for chemicals, including arsenic?
  ☐ Yes ☐ No ☐ Don't know / unsure ☐ Refused
- If it has been tested for arsenic, what were the results?__________________________
  ☐ Don't know / unsure ☐ Refused

13. In the last week, did you visit family or friends who drink or cook with water that comes from a private well?

☐ Yes ☐ No ☐ Don't know / unsure ☐ Refused
- If Yes – did you drink water or eat meals or snacks with those family or friends while you were visiting them?
  ☐ Yes ☐ No ☐ Don't know / unsure ☐ Refused
- If Yes- where do they live? ________________________________
- If Yes – has the water been tested for chemicals, including arsenic?
  ☐ Yes ☐ No ☐ Don't know / unsure ☐ Refused
- If Yes – what were the arsenic results? ________________________________
  ☐ Don't know / unsure ☐ Refused

14. Did you travel within the last week?
    ☐ Yes
    - If Yes – where did you travel?

      ____

    - I'm going to list some international locations. If I mention a location you traveled to in the last week, please tell me.

      ____

      ☐ Chile
      ☐ Argentina
      ☐ Bangladesh
      ☐ India
      ☐ Nepal
      ☐ China
      ☐ Taiwan
      ☐ Thailand
      ☐ Other:______________________________

      ☐ Don't know / unsure
      ☐ Refused

    ☐ No
    ☐ Don't know / unsure
    ☐ Refused

15. Arsenic has been found in some imported herbal medicines and traditional remedies. These kinds of products are sometimes called "homeopathic," "naturopathic", or "folk" medicines. Do you take any of these products, particularly any that have been imported from countries like China or India?
    ☐ Yes ☐ No ☐ Don't know / unsure ☐ Refused
    - If Yes – please name the products (including brand if known) that you take, and approximately how often you take them (*e.g.*, daily, weekly, or monthly):

      ____

      ☐ Don't know / unsure ☐ Refused

16. Arsenic may be found in some seaweed or algae supplements, such as kelp supplements. Do you take any of these types of supplements? (*Note to staff administering survey – ask follow-up questions on serving sizes as needed and fill out data table for questions 16 and/or 17.*)
    ☐ Yes
    - If Yes:
      - Can you tell me the types and brand names of the kelp and/or other seaweed and algae supplements that you take?

        ____

      - For each supplement that you take, approximately how often you take it (*e.g.*, daily, weekly, or monthly)?

        ____

      - For each supplement that you take, how many or how much do you take at a time?

        ____

    ☐ No
    ☐ Don't know / unsure
    ☐ Refused

17. Next I'll ask you for some more information about your daily habits – as I mentioned earlier, just because I am asking this does not mean these items contain arsenic. Do you take any other nutritional or other types of supplements or vitamins regularly? (*Note to staff administering survey – ask follow-up questions on serving sizes as needed and fill out data table for questions 16 and/or 17.*)

☐ Yes

- If Yes:
  - Can you tell me the types and brand names of the supplements that you take?
    ____
  - For each supplement that you take, approximately how often you take it (*e.g.,* daily, weekly, or monthly)?
    ____
  - For each supplement that you take, how many or how much do you take at a time?
    ____

☐ No
☐ Don't know / unsure
☐ Refused

18. This question is about pressure-treated wood. Most types of pressure-treated wood available today do not contain arsenic. However, pressure-treated wood that is older and pressure-treated wood used for certain applications (e.g., for foundations, shingles or utility poles) can contain arsenic. Arsenic-treated wood may be greenish in color, and may have visible small slits where the chemicals were injected. Pressure-treated wood may be found in outdoor structures such as playground equipment, decks, picnic tables, and fences. Do you have regular contact with this type of wood?

☐ Yes ☐ No ☐ Don't know / unsure ☐ Refused

- If Yes – do you know whether the structure was installed before 2004?
  ☐ Yes ☐ No ☐ Don't know / unsure ☐ Refused
- If Yes – do you ever barbecue with or otherwise burn recycled wood (for example in your fireplace)? Some recycled wood may include pressure-treated wood.
  ☐ Yes ☐ No ☐ Don't know / unsure ☐ Refused

19. Some soils can contain elevated levels of arsenic. This is unlikely to be a source of significant arsenic exposure, but to be thorough we want to ask you how much contact you have with soil. Do you regularly participate in activities that involve extensive contact with soil, dirt, or dust? Some examples include gardening, off-roading, or trail hiking. *(Note to staff administering survey - see table for question 19 at the end of this questionnaire to fill in responses.)*

☐ Yes

- If Yes - What activities do you participate in, what locations do you do them in, and approximately how often do you participate in them (*e.g.,* daily, weekly, or monthly)?
  ____
  ☐ Don't know / unsure ☐ Refused

☐ No
☐ Don't know / unsure
☐ Refused

20. Now I'm going to ask you a question related to arts and crafts materials. Do you use specialized art pigments? Some pigments containing arsenic can still be purchased (for example, Orpiment and Conichalcite).

☐ Yes ☐ No ☐ Don't know / unsure ☐ Refused

21. Arsenic has been found in some counterfeit, knock-off, and imported cosmetics, and some cosmetics or personal care products containing muds or herbs. The products containing muds or herbs could be marketed as “natural,” and could include herbal soaps, herbal creams, and facial masks. Do you use any cosmetics or personal care products that are imported, are unusual or knock-off brands, or that contain herbs or muds?
☐ Yes ☐ No ☐ Don’t know / unsure ☐ Refused
   - If Yes – can you tell me the types and brand names of the cosmetics and personal care products that you use, and approximately how often you use them (*e.g.*, daily, weekly, or monthly)?:____________________
     ☐ Don’t know / unsure ☐ Refused

22. A chemotherapy drug called “Trisenox®” contains arsenic trioxide and is used to treat acute promyelocytic leukemia (APL). Do you know if your doctor has ever administered Trisenox to you? This drug is only given by doctors in a hospital or clinic setting and cannot be prescribed for home use.
☐ Yes ☐ No ☐ Don’t know / unsure ☐ Refused
   - If Yes –when did you last receive Trisenox?
     ____
     ☐ Don’t know / unsure ☐ Refused

23. Do you smoke cigarettes?
☐ Yes ☐ No ☐ Used to smoke, but quit ☐ Don’t know / unsure ☐ Refused
   - If Yes – on average, how many cigarettes do you smoke per day?____________
     ☐ Don’t know / unsure ☐ Refused

24. Certain occupational settings or job-related activities may result in arsenic exposures. Do you know if you work with or have come into contact with arsenic at your job?
☐ Yes ☐ No ☐ Don’t know / unsure ☐ Refused
   - If Yes – what type of work do you do?
     ____
     ☐ Don’t know / unsure ☐ Refused
   - If Yes – when were you employed at this job?
     ____
   - ☐ Don’t know / unsure ☐ Refused

25. I am now going to go through a list of industries and work settings. (*Note to staff administering survey – read down left column first, then right column.)* If you regularly work in or are around any of the following, please tell me.

- ☐ Electronics manufacturing
- ☐ Battery or electronics recycling
- ☐ Emergency response, such as firefighting
- ☐ Agriculture (crops/livestock)
- ☐ Landscape maintenance, especially herbicide application
- ☐ Road or pavement work with road reflectors
- ☐ Construction and/or demolition
- ☐ Concrete production
- ☐ Laboratory
- ☐ Glass manufacturing
- ☐ Mining industry
- ☐ Smelter industry
- ☐ Waste incinerator
- ☐ Industrial processing
- ☐ Coal-burning industry

(*Note to staff administering survey: If respondent indicates "yes" to any of the above categories, ask for details on where he/she goes [e.g., facility name, location], what he/she does at the industry/facility [e.g., tend livestock], whether he/she uses personal protective equipment and follows other guidelines, if he/she is aware of any arsenic uses [e.g., herbicides] in these settings, etc. If participant indicates that he/she does road or pavement work with road reflectors, ask if the reflectors are made of glass beads and what is the extent of contact the participant has with them. Please use additional pages as needed.)*

26. Can you think of any other information about your possible current or past arsenic exposures?

____

Thank you for taking this survey. We will contact you by telephone to discuss your potential exposures to arsenic after we review your answers.

DATA TABLE FOR QUESTION 2 (rice products)

Type of rice product	Brand, if identified	Frequency in last 3 days	Amount consumed (serving size)

DATA TABLE FOR QUESTION 3 (foods containing brown rice syrup)

Type of food that may contain brown rice syrup	Brand, if identified	Frequency in last 3 days	Amount consumed (serving size)

DATA TABLE FOR QUESTION 4 (rice-based ingredients)

Type of rice-based ingredient	Food prepared with rice-based ingredient	Brand, if identified	Frequency in last 3 days	Amount consumed (serving size)

DATA TABLE FOR QUESTION 8 (mushrooms)

Foraging location	Type(s) of mushrooms collected

DATA TABLE FOR QUESTION 9 (alcoholic beverages)

Type of beer or wine (including sake [rice wine])	Brand, if identified	Frequency in last 3 days	Amount consumed (serving size)

DATA TABLE FOR QUESTION 10 (tea)

Tea type	Brand and/or type, if identified (e.g., Twinings Earl Grey)	From which country, if identified	Frequency in last 3 days	Amount consumed (serving size)

DATA TABLE FOR QUESTION 11 (apple and grape juice)

Type of juice	Brand, if identified	Frequency in last 3 days	Amount consumed (serving size)

DATA TABLE FOR QUESTIONS 16 and/or 17 (supplements)

Kelp, seaweed, or algae supplement	Brand, if identified	Frequency of consumption	Amount consumed (e.g., teaspoon/ tablespoon; number of pills)
Other supplements	Brand, if identified	Frequency of consumption	Amount consumed

DATA TABLE FOR QUESTION 19 (soil/ dirt/ dust activities)

Activity description	Location(s)	Frequency
